# Challenges and Strategies in Ascribing Functions to Long Noncoding RNAs

**DOI:** 10.3390/cancers12061458

**Published:** 2020-06-03

**Authors:** Yang Zhao, Hongqi Teng, Fan Yao, Shannon Yap, Yutong Sun, Li Ma

**Affiliations:** 1 Department of Experimental Radiation Oncology, The University of Texas MD Anderson Cancer Center, Houston, TX 77030, USA; YZhao18@mdanderson.org (Y.Z.); HTeng1@mdanderson.org (H.T.); FYao@mdanderson.org (F.Y.); SSYap@mdanderson.org (S.Y.); 2 Department of Molecular and Cellular Oncology, The University of Texas MD Anderson Cancer Center, Houston, TX 77030, USA; YSun2@mdanderson.org; 3 UTHealth Graduate School of Biomedical Sciences, The University of Texas MD Anderson Cancer Center, Houston, TX 77030, USA

**Keywords:** lncRNA, in *cis*, in *trans*, CRISPR

## Abstract

Long noncoding RNAs (lncRNAs) are involved in many physiological and pathological processes, such as development, aging, immunity, and cancer. Mechanistically, lncRNAs exert their functions through interaction with proteins, genomic DNA, and other RNA, leading to transcriptional and post-transcriptional regulation of gene expression, either in *cis* or in *trans*; it is often difficult to distinguish between these two regulatory mechanisms. A variety of approaches, including RNA interference, antisense oligonucleotides, CRISPR-based methods, and genetically engineered mouse models, have yielded abundant information about lncRNA functions and underlying mechanisms, albeit with many discrepancies. In this review, we elaborate on the challenges in ascribing functions to lncRNAs based on the features of lncRNAs, including the genomic location, copy number, domain structure, subcellular localization, stability, evolution, and expression pattern. We also describe a framework for the investigation of lncRNA functions and mechanisms of action. Rigorous characterization of cancer-implicated lncRNAs is critical for the identification of bona fide anticancer targets.

## 1. Introduction

The vast majority of the human genomic DNA is transcribed into RNA, of which 2% encodes proteins, while the remaining 98% is recognized as noncoding RNA [1]. Long noncoding RNAs (lncRNAs) are defined as transcripts that are longer than 200 nucleotides and are not translated into proteins [2]. LncRNAs have been demonstrated to play roles in chromatin structure and epigenetic remodeling, regulation of pre-mRNA (messenger RNA) splicing, mRNA translation, and RNA metabolism, as well as protein localization, decay, and activity [3]. Mechanistically, lncRNAs regulate gene expression and/or function, either positively or negatively, by interacting with DNA, RNA, and proteins, and have been shown to modulate transcriptional, post-transcriptional, and post-translational processes. At the transcriptional level, lncRNAs act on target genes either in cis (i.e., targeting neighboring genes) or in trans (i.e., targeting distant genes) [2,4].

The field of lncRNAs has exploded in the past decade. Many new lncRNAs have been identified, annotated, and implicated in various physiological and pathological processes, including tumor growth and metastasis. However, ascribing functions to a specific lncRNA has proven considerably more challenging than initially anticipated. Compared with protein-coding genes, our understanding of lncRNA functions and mechanisms is much more limited, and many caveats and controversies remain, which could be associated with several characteristics of lncRNAs [5,6,7]. First, most lncRNAs are expressed at low levels relative to mRNAs and microRNAs, making it difficult to capture and annotate lncRNAs from transcriptomic data [2]. Second, unlike protein-coding genes with readily identifiable open reading frames (ORFs) that facilitate prediction of the structural domains, 2D or 3D structure, and protein-protein interactions, lncRNAs lack significant ORFs, and the lncRNA sequence-function relationship is poorly understood [2]. Third, unlike microRNAs and mRNAs, most lncRNAs have poor sequence conservation among different species, making it difficult to obtain functional information from evolutionary analyses [8]. In addition, many lncRNAs exhibit unique temporal and spatial expression patterns [8].

LncRNA research is more technically challenging than research on protein-coding genes. Whereas small interfering RNA (siRNA) or short hairpin RNA (shRNA) can be used to knock down most protein-coding genes efficiently, the RNAi (RNA interference) approach is relatively less effective for lncRNAs due to their localization and expression [9,10]. Due to recent advances in genome editing, the CRISPR-Cas9 technology has emerged as a potent and versatile tool to manipulate a variety of genomic elements including lncRNAs [11,12]; however, specific constraints exist. For protein-coding genes, it is usually sufficient to use a single guide RNA (gRNA) to target the ORF, because insertion-deletion (indel) mutations can lead to frameshifts and subsequent depletion of the encoded protein [13]. In contrast, lncRNAs have no ORF, and thus, it is highly unlikely that they can be inactivated in this way. Therefore, modifications of the basic CRISPR-Cas9 approach have been developed to target lncRNAs. These include double-gRNA-mediated deletion [11] and transcriptional modulation (activation or inhibition) [14,15]. In this review, we discuss the features of lncRNAs as well as the challenges and strategies in ascribing functions to lncRNAs. In light of the substantial controversies in the field, a framework for the rigorous characterization of lncRNA functions and mechanisms of action needs to be established for lncRNA research.

## 2. The Genomics of LncRNA Genes

Based on the genomic location with respect to the nearest protein-coding gene, lncRNAs can be divided approximately equally into genic and intergenic classes—genic lncRNA genes overlap with a protein-coding gene locus, whereas intergenic lncRNA genes do not [16]. Based on their position and orientation relative to the overlapping protein-coding gene, genic lncRNAs can be divided into sense versus antisense subgroups or intronic versus exonic subgroups.

Classification by genomic location sometimes gives clues on the regulatory mechanism of the lncRNA. For instance, an antisense lncRNA may regulate its sense counterpart through direct sense-antisense pairing. This is exemplified by a lncRNA elevated in Alzheimer’s disease, *BACE-AS1*, which upregulates BACE expression by binding and stabilizing *BACE* mRNA [17]. Additional antisense lncRNAs, such as *Uchl1-AS* [18], *PDCD4-AS1* [19], and *KRT7-AS* [20], function similarly to promote mRNA stability or translation through base pairing with their sense counterpart. On the other hand, certain antisense lncRNAs downregulate sense gene expression. For example, the lncRNA *GLS-AS* inhibits GLS (glutaminase) expression by forming double-stranded RNA with *GLS* pre-mRNA through a Dicer-dependent RNA interference mechanism, leading to suppression of GLS-mediated metabolism and pancreatic cancer progression [21]. Because of the importance of RNA-RNA interactions in sense-antisense pairing, computational tools, such as MechRNA [22] and IntaRNA 2.0 [23], have been developed to predict the probability of RNA duplex formation based on the calculation of thermodynamic free energy. Moreover, a new sequencing method, named RNA in situ conformation sequencing (RIC-seq) [24], has emerged as an experimental tool to identify RNA-RNA interactions.

In addition to RNA-RNA base pairing, antisense lncRNA genes can also act in cis by interfering with the sense gene transcription, resulting in the inverse correlation between sense and antisense transcripts [25]. This is exemplified by the lncRNA gene *Airn*, which overlaps with the *Igf2r* promoter. This overlap interferes with the recruitment of RNA polymerase II, leading to repression of *Igf2r* gene transcription in cis [26]. A recent study revealed another mechanism by which mRNA transcription promotes the transcription of its antisense lncRNA through an R-loop-dependent mechanism [27]. Moreover, antisense lncRNAs can form RNA-DNA complexes to regulate gene expression or act as the scaffold to assemble protein complexes [28,29,30].

A subgroup of intergenic lncRNAs, called divergent lncRNAs, are transcribed in the opposite direction to nearby protein-coding genes. Divergent lncRNAs and mRNAs are transcribed in a head-to-head fashion, typically less than 1 kb apart [31,32]. In fact, bidirectional promoters are widely prevalent, and bidirectional genes represent more than 10% of all human genes [33,34]. In human and murine embryonic stem cells (ESCs), more than 60% of lncRNA transcripts originate from divergent transcription at promoters of active protein-coding genes (PCGs), and the transcribed lncRNA-mRNA pairs exhibit coordinated changes in transcription during ESC differentiation [35]. Further studies have revealed that many but not all divergent lncRNAs show correlated expression or synergetic effects with their PCG partners, which can be exemplified by *ncRNA-RB1* [33] and *PANDA* [36]. Although the mechanism of this co-regulation is not fully understood, one hypothesis is that the act of transcription itself suppresses negative effects on the promoter, such as the spread of repressive chromatin, permitting steady PCG expression [31]. It has been proposed that divergent transcription may shape the evolution of the genome through the origination of new genes [37].

When investigating lncRNAs, we need to take into account the genomic location and we also should consider the genomic copy-number variation (CNV), especially copy number amplification (CNA)—a common genomic alteration found in human epithelial tumors [38,39]. A valuable tool to study lncRNA genes, the CRISPR-Cas9-mediated genome editing approach, induces double-stranded breaks in DNA by the Cas9 endonuclease in a single guide RNA (sgRNA)-directed sequence-specific manner. This damage can be repaired by nonhomologous end-joining (NHEJ), an error-prone repair method that induces indel mutations [40]. In the case of CNA, there are more sgRNA target sequences, which increases the chance of Cas9-mediated DNA cuts and double-strand break-induced antiproliferative effects [41,42], especially in p53 wild-type cells [43,44,45]. Another issue is that CNA of essential genes may protect them from complete knockout because Cas9-mediated genome editing is likely to be incomplete and because more copies of the target gene may increase resilience to knockout editing [39]. Because of these issues, CNVs may influence CRISPR screening scores, thus generating false-positive hits. For example, Liu et al. [46] performed a genome-wide CRISPR screen by targeting splice sites of lncRNAs to deplete lncRNA expression. Further analysis of the results from this screen revealed that at least 30% of the hits were false positives due to either nuclease activity in copy number-amplified regions or overlap with protein-coding genes [42]. The CRISPR interference (CRISPRi) method, which does not induce DNA cuts, could mitigate this issue [15,41,42]. Thus, it is important to consider the copy number of the lncRNA of interest and to choose the proper CRISPR method to target it [47,48,49].

## 3. LncRNA Structure and Its Link to Functions

RNA adopts various structural elements including the stem-loop, pseudoknot, triplex, and G-quadruplex and is capable of long-range interaction, which contributes to its biological functions [50]. These structural elements can form through intramolecular interactions within the same RNA molecule or intermolecular interactions with other molecules such as other RNA, DNA, and proteins. Identifying RNA motifs, structures, and interactions with binding partners is critical for understanding gene regulation and function [50,51,52,53].

For protein-coding genes, the primary sequence of amino acids can be used to predict functional domains and interactions with other proteins, and proteins with similar functions often share common domains. In contrast to proteins, lncRNAs with similar functions often lack a homologous primary sequence, and the knowledge about the function of one lncRNA hardly informs the functions of other lncRNAs [54]. Nevertheless, it is thought that, like proteins, lncRNAs function through “domains” or discrete elements that mediate molecular interactions and subcellular localization [55].

One of the mysteries about lncRNAs is the discrepancy between low primary sequence conservation and functionally conserved roles [1]. Hence, significant efforts have been dedicated to the search for conserved structural elements. For instance, a large number of correlated positions in lncRNAs were revealed by multiple alignments, suggesting evolutionary conservation of the secondary structures of lncRNAs [8,56]. In addition, many conserved structure elements were found to be enriched in lncRNAs through screening for functional RNA structures conserved between mice and 59 other vertebrates [57]. However, these findings are based on computational predictions. It remains to be seen how many of these predicted conserved structures are real and functionally important in vivo [1].

In addition to computational predictions, experimental support for “domain” prediction and validation for a specific lncRNA is beginning to emerge [58,59,60]. The structural and biological features of some well-known lncRNAs, such as metastasis associated lung adenocarcinoma transcript 1 (*MALAT1)* [61,62,63], nuclear enriched abundant transcript 1 (*NEAT1)* [62], HOX transcript antisense intergenic RNA *(HOTAIR)* [64], and *lincRNA-p21* [65], have been experimentally characterized. Moreover, methods for probing the RNA “structurome,” which couple high-throughput sequencing (HTS) with ribonuclease cleavage or chemical probing, have facilitated the transcriptome-wide mapping of RNA structure [50]. For instance, Fang et al. [66] developed targeted structure-seq, which combines chemical probing of RNA structure in cells with target-specific HTS. By enriching for signals from the RNA of interest, targeted structure-seq achieved high coverage of the full-length *Xist* (X-inactive specific transcript) lncRNA. It should be noted that HTS-based RNA structure profiling technologies rely heavily on the development of robust computational methods. Significant challenges in these methods, such as the need to improve the accuracy, signal-to-noise ratio, and sequencing depth, remain to be overcome.

Recent studies have demonstrated the use of specific elements to predict lncRNA localization and function. Carlevaro-Fita et al. [55] reported that a subset of transposable elements (TEs), otherwise known as repeat insertion domains of lncRNA (RIDLs), undergo evolutionary selection and can drive nuclear enrichment of lncRNAs. TEs act as functional lncRNA domains through two possible mechanisms: (1) TEs are recognized by the cellular transport pathway; (2) TEs destabilize transcripts, causing a concentration gradient from the nucleus to the cytoplasm [55]. Other elements, such as Alu elements and C-rich motifs, both of which interact with the nuclear RNA-binding protein HNRNPK, can increase the nuclear accumulation of lncRNAs in a conserved manner [67]. Thus, while the precise molecular mechanisms remain to be validated, elements like TEs, Alu elements, and C-rich motifs are valuable for predicting lncRNA localization and function.

Another way of predicting lncRNA functions is k-mer-based classification, which is a sequence comparison method designed to deconstruct linear sequence relationships in lncRNAs and assess similarity based on the abundance of short motifs named k-mers (“k” here specifies the length of the motif, varying from three to eight) [54]. K-mer profiles are found to correlate with the protein binding and subcellular localization of lncRNAs. Interestingly, evolutionarily unrelated lncRNAs could exert similar functions through different spatial arrangements of related sequence motifs. Altogether, k-mer-based classification is a useful approach to predict sequence-function relationships in lncRNAs [54].

In Table 1, we summarize the lncRNA motifs and elements that have been implicated in regulating lncRNA functions. The regulations are categorized into three subgroups: regulation of lncRNA stability, regulation of lncRNA localization, and regulation of lncRNA interactions with other molecules.

## 4. The Subcellular Localization and Stability of LncRNAs

Unlike mRNAs, lncRNAs are more abundant in the nucleus than in the cytoplasm [7]. Previous reviews have summarized the relationship between the subcellular location and function of lncRNAs in detail [81,82]. Here we mainly discuss how to predict a lncRNA’s subcellular distribution through its primary manifestations such as its sequence, and how to determine lncRNA’s localization experimentally.

The structure of lncRNA correlates with the subcellular localization. Recently developed HTS-based methods have provided rich information through the analysis of sequence elements such as TEs, Alu elements, and C-rich motifs, as discussed above. A limitation is that the element analysis of the primary sequence may not be fully informative, since the 2D or 3D structure of lncRNA could influence the localization. Methods to predict the localization of lncRNAs by analyzing high-dimension structures in a high-throughput way are still lacking.

The lncRNA localization can be visualized by staining methods, such as fluorescent in situ hybridization (FISH). This method is based on tracking the target RNA with multiple short DNA probes, which are complementary to the target and are conjugated with a fluorescent dye [83]. For imaging intracellular localization of single RNA transcripts in fixed cells or tissues, FISH is still regarded as the gold-standard approach [84,85]. For live-cell imaging of the RNA of interest, stem-loop labeling and fluorescence protein tagging by the MS2-MCP system are useful [86,87]. However, the insertion of dozens of MS2 aptamers into specific lncRNA gene loci makes widespread application of this method difficult, and a common concern is whether such insertions affect RNA structure, dynamics, expression, and function. Recently, CRISPR technology has provided a new approach to determine RNA localization in live cells. The nuclear-localized RNA-targeting Cas9 (RCas9) can be used to visualize highly abundant mRNAs in live cells [88,89], but it is not suitable for most lncRNAs, which are not abundantly expressed. On the other hand, a novel CRISPR-Cas13 system showed feasibility in imaging lncRNAs in real-time with high specificity and sensitivity [82]. Unlike Cas9, Cas13 specifically binds to RNA, not double-stranded DNA. Recent engineering of CRISPR-Cas13 has enabled precise RNA targeting and editing in mammalian cells [90]. Yang et al. [91] compared different bacteria and identified dPspCas13b from Prevotella sp. P5-125 as the most efficient one; they showed that an optimized dCas13 system could label *NEAT1*, *SatIII*, *MUC4*, and *GCN4* RNAs and that combining dCas13 with MS2-MCP or Cas9 achieved dual-color imaging of RNAs or simultaneous visualization of RNA and genomic DNA in living cells [91]. Widespread use of this technology still awaits general guidelines for designing efficient gRNAs targeting a lncRNA of interest. For effective gRNA targeting, the structure and conformation of the targeted lncRNA should be considered.

In addition to FISH and CRISPR, novel high-throughput methods have been developed to detect lncRNA distribution. For instance, Yin et al. [73] used a random, mutagenesis-coupled, high-throughput method called RNA elements for subcellular localization by sequencing (mutREL-seq) to identify an RNA motif that recognizes the U1 small nuclear ribonucleoprotein (snRNP) and is critical for the localization of RNAs to chromatin. Moreover, predictive models, based on systematic analyses of deep sequencing results including expression levels, splicing, gene architecture, chromatin marks, and sequence elements, can be an alternative way of predicting lncRNA localization [92]. Notably, many lncRNAs have both nuclear and cytoplasmic fractions, and they have different functions and mechanisms of action in different subcellular compartments. For example, when localized in the cytoplasm, the lncRNA *MEG3* interacts with PTBP1 (polypyrimidine tract binding protein 1) to promote mRNA decay [93]; when in the nucleus, *MEG3* forms RNA-DNA triplex structures to regulate genes in the TGF-β pathway [94].

In addition to subcellular localization, lncRNA stability should also be considered when investigating lncRNA functions. In systematic surveys, Clark et al. [95] used a custom noncoding RNA array to analyze the half-lives of approximately 800 lncRNAs and 12,000 mRNAs in the mouse Neuro-2a cell line, and Tani et al. [96] developed a 5′-bromo-uridine immunoprecipitation chase-deep sequencing (BRIC-seq) method, which involves pulse-labeling of endogenous RNAs with 5-bromo-uridine and measuring the decrease in RNA levels over time through sequencing. These studies revealed that lncRNA stability varies over a wide range, and the half-life of a significant set of short-lived lncRNAs is regulated by external stimuli.

Notably, lncRNA stability correlates with localization and could to some degree inform the regulatory function [96]. Tracking the subcellular localization of lncRNAs revealed widespread trafficking to different cellular locations, with nuclear lncRNAs more likely to be unstable [95,97]. In fact, nuclear transcripts are generally less stable [8,98]. The fast turnover of short-lived nuclear lncRNAs renders these transcripts less likely to leave the chromatin through a U1 snRNP-mediated mechanism [73,99]. The coupling between the chromatin association and instability of lncRNAs may contribute to the in-cis regulatory function of lncRNAs. Indeed, most short-lived lncRNA transcripts spread locally within their neighborhoods, whereas a few stable and abundant lncRNAs, such as *MALAT1*, exist long enough to be trans-targeted to other sites. For stable lncRNAs, persistent binding with U1 snRNP may drive lncRNA mobilization to distinct nuclear compartments (such as nuclear speckles) or distant genomic sites (in the case of *MALAT1*) [73].

## 5. Evolutionary Conservation and Tissue Specificity of LncRNAs

High-throughput sequencing (HTS) analysis across species has revealed that certain lncRNA elements, such as lncRNA gene promoters, have a similar level of conversation to mRNA promoters in humans and mice [8,100]. Splicing motifs are also evolutionarily retained in many lncRNAs across species [101]. LncRNA exons are much more conserved than are neutrally evolving ancestral repeat sequences, albeit at much lower levels than protein-coding genes [8].

LncRNA expression is often species-specific. An across-mammalian-genomes analysis showed that 30% of lncRNA transcripts (*n* = 4546) are primate-specific (human, chimp, orangutan, macaque, and marmoset) [8]. A total of 0.7% of lncRNAs are specific to the human lineage, and ~1% of lncRNAs are expressed in all of the 18 species analyzed. Aside from species specificity, lncRNA expression is often tissue type- and cell type-specific—11% of lncRNAs (versus 65% of mRNAs) are detected in all human tissues; 21% of lncRNAs are not detected in any human tissue, and 11% are detected only in a single tissue type [8]. Tissue-type specificity analysis showed that lncRNAs are particularly more specific in the brain, testis, and stem cells [102,103,104,105]. LncRNA expression is also context- and time-dependent. Specific lncRNAs are expressed at different stages of immune cell differentiation and drive the process [4]. For instance, *Lnc-DC* is responsible for the differentiation from myeloid progenitor cells or monocytes into dendritic cells [106]. Also, some lncRNAs have been shown to regulate the activation or inactivation of immune cells, e.g., *Nest* [107] promotes T cell activation, whereas *NRON* [108,109] restricts excess activation.

Comparative analysis requires a set of genes that can be compared as well as the algorithms for matching and similarity evaluation. This is challenging for lncRNAs, because (1) lncRNAs in species other than human and mouse are poorly annotated. (2) LncRNAs lack long regions with high sequence conservation needed for comparison. A lncRNA conserved between human and mouse typically has only 20% inter-species homology, and this drops to 5% when comparing human and fish [110]. Moreover, even for lncRNAs expressed in the same tissue among species, the conservation rate is still low. Comparison of lncRNA expression in the livers of mice, rats, and humans revealed that only 60% and 27% of lncRNAs expressed in mice have homologs expressed in rats and humans, respectively, suggesting that lncRNAs are rapidly evolving in the same tissue across species [111]. (3) Experimental evidence for lncRNAs acting through specific structures is limited; notable exceptions are the Rep A repeat in *XIST* [112,113] and the triple-helix that stabilizes the 3′ ends of *MALAT1* and *NEAT1_2* [63,68,114]. Overall, lncRNAs are less conserved and more species-specific than are protein-coding genes.

It should be noted that conservation can be defined at several levels based on the sequence, structure, and subcellular location. A recent review suggested four classifications of lncRNAs according to these characteristics, specifically Class I (conserved exonic structure), Class II (conserved sequence), Class III (positionally conserved), and Class IV (not conserved) [115]. Class I lncRNAs, whose exon-intron structures and sequences are conserved among species, constitute a minority of conserved lncRNAs, including *MALAT1* and *NEAT1*. LncRNAs in this class tend to have more conserved functionality and less tissue specificity among different species; they are highly expressed relative to other classes and often act in trans. Class III lncRNA genes, which are conserved in the act of transcription of the specific region without sequence or structure conservation, are more likely to function *in cis* through the act of transcription rather than through the mature RNA transcript [115].

Long non-coding RNAs can also be functionally conserved through the following manifestations [115]: (1) loss of homologous lncRNAs results in the same phenotype; (2) homologous lncRNAs function through the conserved mechanism; (3) target genes regulated by lncRNAs are the same; and (4) the loss of function in one species can be rescued by exogenous expression of the homolog from a different species. Experimentally validated examples include *XIST*, which has a key role in X inactivation in both humans and mice [116], and *NEAT1*, which is essential for paraspeckle formation across species [117,118]. On the other hand, do homologous lncRNAs across species always have the same function? Through systematic high-throughput analysis of sequence and positionally conserved lncRNAs across species, Guo et al. [119] discovered the distinct subcellular localization and function of the lncRNA *FAST* (FOXD3 anti-sense transcript 1) in human and mouse embryonic stem cells (ESCs). In human ESCs, *FAST* is mainly expressed in the cytoplasm, where it blocks the interaction of the ubiquitin ligase β-TrCP (transducin repeats-containing protein) with phosphorylated β-catenin, leading to the prevention of β-catenin degradation, activation of WNT signaling, and maintenance of pluripotency. In contrast, mouse *Fast* is nuclear retained in ESCs, and its processing is inhibited by the splicing factor PPIE (peptidylprolyl isomerase E), which is abundantly expressed in mouse ESCs but not in human ESCs [119]. As another example, the lncRNA *HOTAIR* has been reported to have different functions in humans and mice: whereas *HOTAIR* regulates the expression of the *HOXD* cluster in primary human fibroblasts [120], *Hoxd* expression is not affected in mice with deletion of the *Hoxc* cluster encompassing *Hotair* [121]. Furthermore, different groups have reported contradictory results in phenotypes and *Hoxd* expression in *Hotair*-knockout mice [122,123], and this discrepancy remains to be resolved [124]. In sum, when studying a specific lncRNA, it is important to take into account the conservation among species and the lncRNA localization and expression patterns among tissue types.

For investigating cancer-implicated lncRNAs, it is important to consider cancer-type specificity [125,126]. Yan et al. [125] performed a comprehensive analysis of 5037 tumor samples across 13 cancer types from The Cancer Genome Atlas and found that compared with protein-coding genes, the expression and dysregulation of lncRNAs are highly cancer type-specific and are characterized by tumor tissue type-specific lncRNA alterations such as somatic copy number alterations, promoter hypermethylation, and cancer-associated single-nucleotide polymorphisms (SNPs). Some of the lncRNAs that are deregulated in cancer have been functionally characterized. For instance, Hu et al. [127] combined copy-number alteration analysis and loss-of-function screening to identify an oncogenic lncRNA, *FAL1* (focally amplified lncRNA on chromosome 1), that is amplified in ovarian cancer. Interestingly, *FAL1* lncRNA interacts with the epigenetic regulator BMI1 (B cell-specific Moloney murine leukemia virus integration site 1) to repress its target genes including *CDKN1A* (encoding p21) [127]. SNPs associated with cancer risk may involve cancer-driver lncRNAs, as exemplified by *CCAT2* (colon cancer associated transcript 2), a lncRNA that encompasses the rs6983267 SNP and is overexpressed in microsatellite-stable colorectal cancer [128]. The SNP rs6983267 has two allelic forms containing either T or G, with the G allele associated with a higher risk for colorectal cancer than the T allele [128]. Altogether, genomic and pan-cancer analyses of lncRNAs have provided valuable information about cancer-driver lncRNAs and their potential use in cancer classification, diagnosis, prognosis, risk assessment, and treatment [125,129,130,131,132].

## 6. LncRNA Functions: In Cis versus In Trans

**Figure 1:**
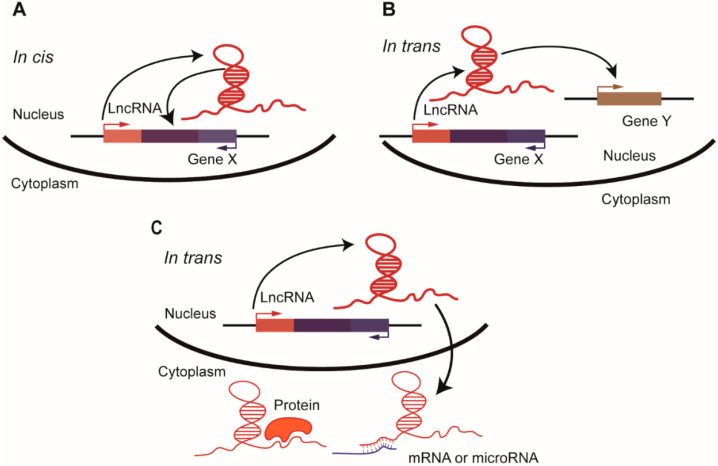
The modes of action of long non-coding RNAs (lncRNAs) can be in cis or in trans. (**A**) Cis-acting lncRNAs regulate the transcription of their neighboring genes. The in-cis regulation can be mediated by the transcribed lncRNA molecule (as illustrated here), the act of transcription or splicing, or the regulatory DNA elements within the lncRNA gene. Representative lncRNAs in this category include *Airn*, *Bendr*, *Blustr*, and *lincRNA-p21*. (**B**) In the nucleus, trans-acting lncRNAs leave the site of transcription and regulate the chromatin states and transcription of genes located in distant regions; some lncRNAs modulate nuclear structures such as nuclear speckles and paraspeckles. Representative lncRNAs in this category include *MALAT1*, *NEAT1*, *HOTAIR,* and *FAL1*. (**C**) Some trans-acting lncRNAs are exported from the nucleus to the cytoplasm, where they bind proteins or other RNAs to regulate the activities of their binding partners. Representative lncRNAs in this category include *FAST*, *PVT1*, and *NORAD*.

The regulatory functions of lncRNAs can be classified broadly as in-cis regulation and in-trans regulation [2] (Figure 1). Notably, some cis-acting lncRNA genes do not act in a sequence-specific manner; instead, the act of transcription or the DNA element within the lncRNA gene locus is more likely to be the root of the regulatory activity than is the lncRNA transcript [133]. Thus, cis-acting lncRNAs have at least three possible mechanisms of action [2]—(1) the lncRNA molecule regulates the transcription of its adjacent genes by recruiting proteins or molecular complexes to the locus or by modulating their activities; (2) the process of transcription or splicing of the lncRNA affects its neighboring gene expression in a sequence-independent manner; and (3) the DNA elements within the lncRNA gene act as regulatory sequences for nearby genes.

It is challenging to distinguish the activity of the lncRNA molecule from that of the DNA where it is transcribed. By using a genome-editing approach in hybrid mouse ESCs, Engreitz et al. [132] found that the deletion of five of 12 lncRNA gene promoters altered the expression of adjacent genes. By terminating transcription with early polyA sequences, Engreitz et al. [132] showed that these cis-regulatory effects were independent of the transcription of the lncRNA locus beyond the first 1–3 exons. For instance, the deletion of the promoter of the lncRNA gene *Bendr*, but not the insertion of a transcriptional terminator into the first intron of *Bendr*, downregulated the expression of the adjacent protein-coding gene *Bend4*. These results demonstrate that lncRNA loci can regulate local gene expression through DNA elements, which is independent of the lncRNA transcripts [132].

In addition to acting in cis, lncRNAs can leave the site of transcription and function in trans. There are three major subgroups of these lncRNAs [2]: (1) lncRNAs that regulate chromatin states and transcription of genes located in distant regions; (2) lncRNAs that modulate nuclear structure and organization; and (3) lncRNAs that bind proteins or other RNAs to regulate their activities. A number of cancer-implicated lncRNAs, including *FAL1* [127], *CCAT2* [128], *LINP1* (LncRNA In Non-homologous End Joining Pathway 1) [133], and *MALAT1* [134], exert their functions through interaction with proteins. In addition, lncRNAs are also capable of binding lipids. *LINK-A* (Long Intergenic Non-coding RNA for Kinase Activation), an oncogenic lncRNA in breast cancer, directly binds phosphatidylinositol-(3–5)-trisphosphate (PIP_3_) to promote its interaction with AKT (protein kinase B) and subsequent AKT activation [135]. Moreover, *LINK-A* facilitates the crosstalk between PIP_3_ and the inhibitory G-protein-coupled receptor (GPCR), leading to downregulation of classic tumor suppressors and the antigen peptide-loading complex [136]. Some lncRNAs can form a feedback loop with their target to promote oncogenic signaling. For example, *PVT1* (plasmacytoma variant translocation 1) is a well-known lncRNA that is amplified or overexpressed in cancer. Both *MYC* and *PVT1* are located on chromosome 8q24, a region amplified in cancer. Upon phosphorylation on threonine 58 (Thr58), *MYC* protein is destabilized and degraded by proteasomes. The lncRNA *PVT1* promotes *MYC* stability by inhibiting its Thr58 phosphorylation. *MYC* protein, in turn, translocates into the nucleus and interacts with the *PVT1* promoter to activate its transcription [137,138,139].

Loss-of-function studies of trans-acting lncRNAs should include genetic rescue experiments, in which the lncRNA is re-expressed from an independent transgene, to distinguish RNA-specific effects from those arising from manipulation of the underlying genomic DNA or other non-specific effects. *MALAT1* is among the most conserved lncRNAs and is highly abundant in normal tissues [140]. Recently, Kim et al. [134] used a transcriptional terminator insertion strategy [141] and found that targeted inactivation of the *MALAT1* gene in a transgenic mouse model of breast cancer, without altering the expression of its adjacent genes, promoted lung metastasis; importantly, this phenotype was reversed by genetic add-back of *MALAT1*. Similarly, double gRNA- and CRISPR-Cas9-mediated focal deletion of *MALAT1* in human breast cancer cells, which did not affect *MALAT1′*s neighboring gene expression, induced the ability of these cells to metastasize to the lung, which could be reversed by re-expression of *MALAT1* [134]. Conversely, overexpression of *MALAT1* suppressed breast cancer lung metastasis in transgenic, xenograft, and syngeneic models. Mechanistically, Kim et al. [134] found that *MALAT1* lncRNA binds, sequesters, and inactivates the pro-metastatic transcription factor TEAD (TEA domain), preventing TEAD from associating with its co-activator YAP (Yes-associated protein) and target gene promoters. Unlike previous *MALAT1* studies that used large genomic deletions or antisense oligonucleotides (ASOs) without rescue experiments [142], Kim et al. [134] used targeted insertional inactivation and focal deletion approaches, both with genetic rescue experiments to rule out non-specific effects [143]. The findings by Kim et al. [134] demonstrate that *MALAT1* is a metastasis-suppressing lncRNA rather than a metastasis promoter in breast cancer, raising caution about targeting *MALAT1* as an antimetastatic strategy. Furthermore, the work by Kim et al. [134] suggests that the previously observed upregulation of *Malat1′*s 12 adjacent genes upon *MALAT1* gene deletion [142] was not due to the loss of *MALAT1* RNA.

When assessing in-cis versus in-trans regulation, it is important to take into account the copy number of the lncRNA. A key question is whether the lncRNA of interest is abundant enough to exert the proposed effect, especially when its target is highly expressed. Thus, when considering the stoichiometric interaction, it is imperative to know the copy numbers of the lncRNA and its targets to establish the plausibility of the regulatory mechanism. For instance, the lncRNA *NORAD* has been identified as a molecular decoy of the RNA-binding proteins PUM1 and PUM2 [76]. PUM proteins bind the 3′ UTR of mRNAs to regulate target mRNA translation and decay. PUM-binding RNAs contain a conserved sequence, UGUANAUA, termed the PUMILIO response element (PRE) [144]. *NORAD* is an abundantly expressed lncRNA and each *NORAD* transcript has 15 PRE sequences. In cells, this lncRNA is sufficient to occupy PUM1 and PUM2 proteins, and the stoichiometric interaction can explain how *NORAD* competes against PUM proteins’ target mRNAs and inhibits their ability to bind other RNAs [76,77]. Another example is *lincRNA-p21*. Initially, RNAi experiments suggested that *lincRNA-p21* represses gene transcription globally in trans [145]; however, a knockout mouse model, generated by focal deletion of the promoter and the first exon, revealed a role for *lincRNA-p21* in activating p21 expression in cis [146]. In light of the short half-life (<2 h) and low copy number (8 copies per cell) of *lincRNA-p21* [146,147], it is unlikely that this lncRNA has a global in trans effect on the genome.

## 7. Strategies for Studying LncRNA Functions

**Figure 2:**
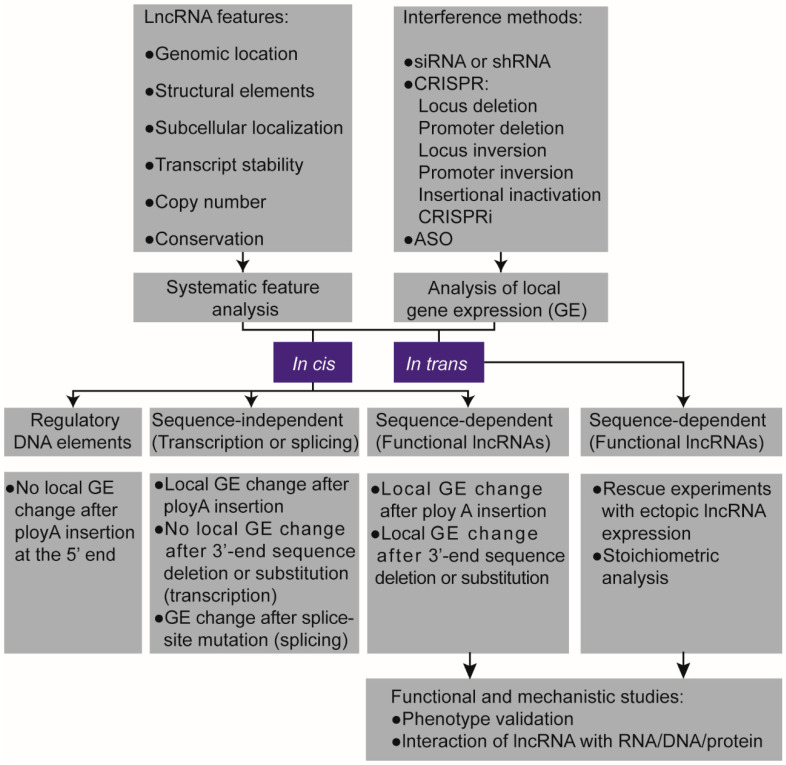
Framework for the functional characterization of lncRNAs. CRISPR: Clustered regularly interspaced short palindromic repeats. ASO: antisense oligonucleotide, lncRNA: long non-coding RNA, siRNA: small interfering RNA, shRNA: short hairpin RNA.

**Figure 3:**
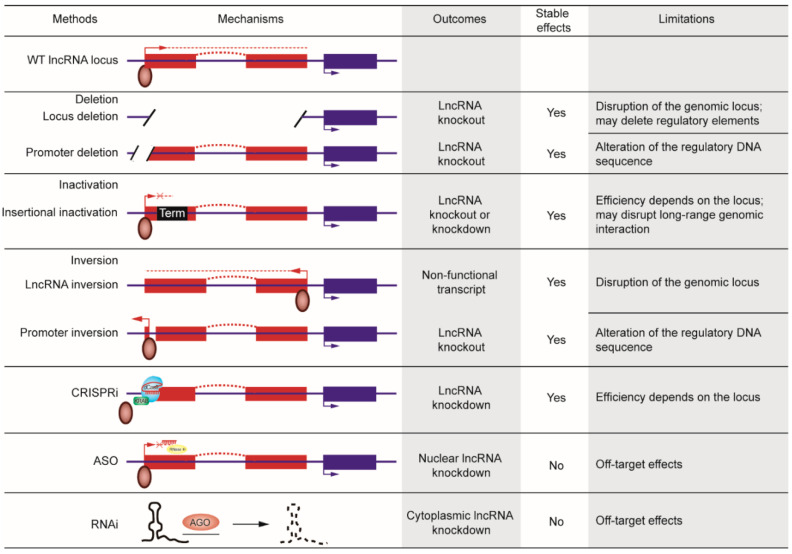
Loss-of-function approaches for investigating lncRNAs. Dark red rectangles: lncRNA exons. Dark blue rectangles: neighboring protein-coding genes. Brown ovals: transcriptional factors. Arrows: the direction of transcription. Black rectangle: transcriptional terminator (Term). Antisense oligonucleotides (ASOs) are illustrated by a complementary sequence to the nascent RNA transcript, which decreases lncRNA expression by triggering RNase H (yellow oval)-mediated cleavage. CRISPRi employs sgRNA-mediated targeting of dCas9 (light blue oval) to the region near the transcriptional start site to repress gene expression, and the repression is enhanced by fusing Cas9 with a repressor, such as the Kruppel-associated box (KRAB) domain (green rectangle). RNAi is triggered by short RNA species that bind to argonaute protein (Ago), which forms a complex to recognize complementary mRNA and lncRNA molecules and induce their degradation.

Considering the features of lncRNAs and the challenges in lncRNA research mentioned above, we recommend a framework for the characterization of lncRNAs (Figure 2), with specific examples. First, we can obtain the annotation of the specific lncRNA through sequence analysis and rule out the protein-coding potential by using algorithms such as PhyloCSF [148]. Next, we can evaluate the likely regulatory mechanism of the lncRNA, in cis or in trans, by analyzing the genomic location, copy number, domain structure, subcellular localization, stability, evolution, and expression pattern as mentioned above. Then, we can examine the expression levels of adjacent genes upon lncRNA depletion, which could be achieved by RNAi, ASOs, and CRISPR-based methods including promoter deletion, entire locus deletion, insertional inactivation (by knocking in a transcriptional terminator), or CRISPRi. The mechanisms, outcomes, and limitations of these methods are summarized in Figure 3. It should be noted that the CRISPR approach has potential drawbacks [42,149,150] and that CRISPR itself may affect local gene expression. For lncRNA genes with copy number amplification, CRISPR-mediated locus deletion might not be suitable because of the increased chances of Cas9-induced DNA cuts and double-strand breaks [42]. For divergent lncRNAs such as *Uph* [151] and *BISPR* [152], CRISPR-mediated promoter deletion should be avoided because of the influence on gene transcription in the opposite direction.

For *cis*-acting lncRNA genes that function in an RNA-independent manner, loss-of-function phenotypes would be seen after deletion of the promoter or specific regulatory DNA elements in the lncRNA gene locus, but not upon the deletion of downstream exons and introns. This is exemplified by *linc1319* (or *Blustr*), which regulates the expression of its neighboring gene *Sfmbt2 in cis* [132]. Promoter deletion or insertion of polyA signals that prematurely terminates transcription, or mutation of the first 5′ splice site of *Blustr*, resulted in significant downregulation of *Sfmbt2*. However, sequential deletions of downstream exons and introns of the *Blustr* gene did not affect *Sfmbt2* expression, suggesting that its *cis*-activating effect is not dependent on its RNA transcript [132].

For *trans*-acting lncRNAs, ASOs have been used to interfere with lncRNA expression in addition to CRISPR-based gene-editing methods. ASOs include gapmers, duplex RNAs, and locked nucleic acids [52]. The RNA-DNA duplex triggers RNase-H-dependent cleavage of transcripts, leading to lncRNA knockdown [153]. This mechanism occurs mainly in the nucleus, making it useful for targeting nuclear lncRNAs that are not amenable to siRNA- or shRNA-mediated silencing. The use of ASOs to knock down lncRNAs has been tested in cancer models, with reported inhibitory effects on tumor growth and progression [154]. However, in addition to the intended target RNA, ASOs may also bind to partially complementary target sites, leading to degradation of unintended RNAs [153]. Moreover, two recent back-to-back studies, from the Mendell group and Ionis Pharmaceuticals, respectively, reported that ASOs cleave nascent RNAs and serve as natural triggers for transcriptional termination mediated by the exonuclease XRN2 [155,156]. A strategy to overcome this side effect is to design ASOs that target the 3′ end of the lncRNA transcript, which allows knockdown of the lncRNA while minimizing the effect on Pol II-dependent transcription [155].

Notably, certain lncRNAs have opposing functions to their genomic DNA. Yin et al. [157] reported opposite effects from the lncRNA *Haunt* gene deletion and insertional inactivation: whereas insertional inactivation of *Haunt* with minimal disruption of its genomic locus led to upregulation of *HOXA* genes, large genomic deletion of the *Haunt* locus attenuated *HOXA* expression. Mechanistically, the *Haunt* gene locus contains enhancers of *HOXA*, whereas *Haunt* RNA acts to downregulate *HOXA* expression. These findings indicate that the *Haunt* gene deletion effect can be attributed to the loss of the genomic DNA, which dominated the effect of *Haunt* RNA loss.

In addition to *Haunt* and *lincRNA-p21* discussed above, accumulating evidence has demonstrated substantially different or opposite phenotypes resulting from different strategies for inactivating the same lncRNA. In mice, genetic deletion of the lncRNA gene *Fendrr* resulted in defects in the lung and gastrointestinal tract [158], whereas transcriptional terminator insertion led to defects in the heart and body wall, which could be rescued by a *Fendrr* transgene [159]. Moreover, RNAi experiments suggested that the lncRNA *Evf2* is important for activating *Dlx5/6* expression [160], but transcriptional terminator insertion in mice showed the opposite effect [161], which could be rescued by an *Evf2* transgene [162]. A recent study characterized the off-target effects of three common loss-of-function methods at the whole-transcriptome level [163]. All three methods, RNAi, ASOs, and CRISPRi, had off-target effects in gene expression. Intriguingly, the various methods each yielded distinct sets of differentially expressed genes as well as different cellular phenotypes. These discrepancies have been observed for the lncRNA *MALAT1*, the protein-coding gene *Ch-TOG/CKAP5*, and a previously uncharacterized lncRNA [163]. Collectively, these findings support the existence of method-specific off-target effects and underscore the importance of alleviating such effects.

## 8. Conclusions

Despite the explosion of publications on lncRNAs in recent years, many caveats and controversies remain in the field. Here, we have discussed the current challenges and experimental strategies in ascribing functions to lncRNAs. Loss-of-function (LOF) approaches, such as RNAi, ASOs, and CRISPR-based genome editing, have proven valuable for studying the biological functions of lncRNAs in normal physiology and cancer. As elaborated above, each method has its limitations. A major concern is non-specific targeting, which has been shown to account for false results in anticancer target identification and characterization. Such off-target effects can be mitigated by (1) analyzing the features of the lncRNA of interest to assess the likelihood of in-cis versus in-trans regulation; (2) using multiple carefully controlled LOF approaches to determine if the LOF effects (including the effect on adjacent gene expression) are consistent; (3) complementing LOF approaches with gain-of-function approaches; and (4) performing genetic rescue experiments.

## Figures and Tables

**Table 1:** List of long noncoding RNA (lncRNA) motifs and elements.

Motifs and Elements	LncRNAs	Functions or Characteristics	References
**Regulation of stability**			
Highly conserved triple helical structure at the 3′ end	*MALAT1* *NEAT1_2*	Critical for protecting the 3′ end of *MALAT1* from 3′–5′ exonucleases; promoting lncRNA stability	[63,68,69]
Alu element	*lncRNA_AF087999*	Base-pairing between the Alu element of a lncRNA and the Alu element of the 3′ UTR of mRNA can create a STAU1-binding site, which regulates mRNA decay	[70]
**Regulation of localization**			
AGCCC motif	*BORG*	Critical for nuclear localization of *BORG*. The binding partner of the motif and the specific mechanism are unknown	[60]
156-bp repeating RNA domain (RRD)	*FIRRE*	Mediates the interaction with hnRNPU to regulate *FIRRE* nuclear localization	[71]
SINE-derived nuclear RNA localization element	*JPX*, *PVT1*	Binds the RNA-binding protein HNRNPK to promote nuclear localization of the lncRNA	[67]
C-rich motif	Multiple lncRNAs	Enriched in nuclear transcripts	[67,72]
U1 snRNA binding site	*MALAT1*	U1 snRNA binds extensively to *MALAT1* lncRNA via U1 snRNP recognition sites to retain *MALAT1* on the chromatin	[73]
Transposable element (TE), otherwise known as RIDL (repeat insertion domain of lncRNA)	*RP11-5407*, *LINC00173*, *RP4806M20.4*	Certain TEs/RIDLs, such as L2b, MIRb, and MIRc, promote the nuclear enrichment of lncRNAs, while GC-rich RIDL elements correlate with cytoplasmic localization of lncRNAs	[55,74]
Short interspersed nuclear element (SINE)	*MALAT1*	SINE deletion leads to the export of *MALAT1* to the cytoplasm	[59]
**Regulation of interaction**			
A-repeat element	*XIST*	Interacts with Spen protein to mediate transcriptional silencing; critical for X chromosome inactivation	[75]
Conserved short sequence motif	*LINC-PINT*	Mediates the interaction with PRC2 and contributes to PRC2-dependent silencing	[58]
PUMILIO response element	*NORAD*	Competes against the target mRNAs of PUMILIO proteins	[76,77]
Conserved androgen receptor (AR)-binding motif	*SLNCR1*, *HOTAIR*, *PCGEM1*	Required for the AR-lncRNA association and regulates the transcriptional activity of AR	[78,79,80]

*MALAT1*: Metastasis Associated Lung Adenocarcinoma Transcript 1. *NEAT1*: Nuclear Enriched Abundant Transcript 1. UTR: untranslated region. STAU1: Staufen Double-Stranded RNA Binding Protein 1. *BORG*: BMP/OP-Responsive Gene. *FIRRE*: Functional Intergenic Repeating RNA Element. *JPX*: Just Proximal to *XIST*. *PVT1*: Plasmacytoma Variant Translocation 1. hnRNPU: Heterogeneous Nuclear Ribonucleoprotein U. HNRNPK: Heterogeneous Nuclear Ribonucleoprotein K. snRNP: small nuclear ribonucleoprotein. TE: transposable element. RIDL: repeat insertion domain of lncRNA. SINE: short interspersed nuclear element. *XIST*: HOX Transcript Antisense Intergenic RNA. *LINC-PINT*: Long Intergenic Non-protein Coding RNA, p53 Induced Transcript. *NORAD*: Non-coding RNA Activated by DNA Damage. *SLNCR1*: SRA-Like Non-Coding RNA 1. *HOTAIR*: HOX Transcript Antisense RNA. *PCGEM1*: Prostate Cancer Gene Expression Marker 1. AR: androgen receptor.
